# Patterns of Nucleotide Diversity at Photoperiod Related Genes in Norway Spruce [*Picea abies* (L.) Karst.]

**DOI:** 10.1371/journal.pone.0095306

**Published:** 2014-05-08

**Authors:** Thomas Källman, Stéphane De Mita, Hanna Larsson, Niclas Gyllenstrand, Myriam Heuertz, Laura Parducci, Yoshihisa Suyama, Ulf Lagercrantz, Martin Lascoux

**Affiliations:** 1 Department of Ecology and Genetics, Evolutionary Biology Center Uppsala University, Uppsala, Sweden; 2 UMR Interactions Arbres-Microorganismes, INRA Nancy, France; 3 Dept. of Plant Biology and Forest Genetics, Swedish Agricultural University, Uppsala, Sweden; 4 Forest Research Centre INIA-CIFOR Madrid, Spain; 5 Graduate School of Agricultural Science, Tohoku University, Osaki, Japan; University of Umeå, Sweden

## Abstract

The ability of plants to track seasonal changes is largely dependent on genes assigned to the photoperiod pathway, and variation in those genes is thereby important for adaptation to local day length conditions. Extensive physiological data in several temperate conifer species suggest that populations are adapted to local light conditions, but data on the genes underlying this adaptation are more limited. Here we present nucleotide diversity data from 19 genes putatively involved in photoperiodic response in Norway spruce (*Picea abies*). Based on similarity to model plants the genes were grouped into three categories according to their presumed position in the photoperiod pathway: photoreceptors, circadian clock genes, and downstream targets. An HKA (Hudson, Kreitman and Aquade) test showed a significant excess of diversity at photoreceptor genes, but no departure from neutrality at circadian genes and downstream targets. Departures from neutrality were also tested with Tajima's D and Fay and Wu's H statistics under three demographic scenarios: the standard neutral model, a population expansion model, and a more complex population split model. Only one gene, the circadian clock gene PaPRR3 with a highly positive Tajima's D value, deviates significantly from all tested demographic scenarios. As the PaPRR3 gene harbours multiple non-synonymous variants it appears as an excellent candidate gene for control of photoperiod response in Norway spruce.

## Introduction

The identification of genetic variants that underlie adaptive traits is one of the long-term goals of evolutionary genetics. In many temperate plant species the presence of adaptation is supported by both physiological and genetic data. For example, transplant studies in *Arabidopsis thaliana* (Arabidopsis) have provided evidence for local adaptation in response to both temperature and light conditions [1], [2]. Photoperiod is of particular importance to plants in temperate regions of the world as it allows them to track seasonal changes without relying solely on temperature, which can vary considerably between years, and initiate appropriate physiological responses. The plants ascertain the change in photoperiod by perceiving the length of day and night over a 24-hour period and integrating these signals with the internal circadian clock. So far, our knowledge on the molecular basis of plant response to photoperiod stems mainly from detailed studies of the model plant Arabidopsis. Genes involved in this response are commonly assigned to the photoperiod pathway and include light receptors, circadian clock genes and downstream targets of these genes. Light receptors such as the phytochromes (PHYA, PHYB, PHYC and PHYD) and the cryptochromes (CRY1, CRY2) and ZEITLUPE (ZTL) are used to capture different parts of the light spectrum, the former being most sensitive to red and far-red light and the latter more sensitive to blue light [3], [4]. These genes, together with integrating factors and other helper molecules, transfer the light signal to the circadian clock and light-regulated target genes. The circadian clock itself consists of a number of interconnected feedback loops that together create an internal rhythm of approximately 24 h length. Key genes here include the pseudo response regulators (ARABIDOPSIS PSEUDO RESPONSE REGULATOR 1-9, [APRR1, APRR3, APRR5, APRR7, APRR9]) and two genes with MYB domains (CIRCADIAN CLOCK ASSOCIATED 1, CCA1 and LATE ELONGATED HYPOCOTYL, LHY) [5]. In Arabidopsis, functional studies have also revealed that the genes GIGANTEA (GI), EARLY FLOWERING 3 (ELF3) and EARLY FLOWERING 4 (ELF4) are required to obtain a stable circadian clock, but their role is somewhat less well defined [6]–[8]. Finally, the signals from light receptors and the circadian clock (as well as other pathways) are integrated into several downstream genes such as CONSTANS (CO) and FLOWERING LOCUS T (FT) that either induce or repress flowering [9]. As data is accumulating from other species, it has become clear that many of the genes involved in photoperiodic response in model plants have a conserved function even in distantly related plant species, including gymnosperm species, like Norway spruce [10], [11]. Further, studies of perennial plants suggest that the photoperiodic response and associated genetic pathways are not only involved in transition to flowering, but also in the control of annual growth, for instance the control of growth cessation in the autumn [12], [13]. We would therefore expect variation at these genes to be associated to variation in fitness.

In population genetic studies aiming at describing the genetic variants underlying local adaptation, a first step has often been to identify genomic regions that display polymorphism deviating from expectations from the standard neutral model (SNM) of evolution. However, in most cases where multilocus data is available, it has become clear that the overall pattern of diversity does not fit the SNM and that ignoring this can lead to false inference of selection. A departure from the SNM has been reported in a number of European forest tree species, where inferences from multilocus sequence data suggest that the species went through severe and ancient bottleneck events followed by population expansion [14]–[16]. This likely reflects range expansion after periods of less suitable climate, when the trees were present in more restricted refugial areas.

The distribution range of Norway spruce [*Picea abies* (L.) Karst.) can be divided into a Nordic-Baltic group covering the entire Fennoscandia and extending to the Urals and a southern Alpine group covering different regions along the mountain ranges of central and southeastern Europe. The present day population genetic structure of Norway spruce is largely accounted for by these two major groups: the between groups 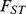 is around 0.10 whereas within groups, between population 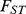 is generally less than 0.05 [14]. Analyses of isozymes, organelle DNA and fossil data suggested the presence of three main spruce refugia during the Last Glacial Maximum (LGM, 22–18,000 years ago) [17]–[20]. A recent study proposed survival of spruce populations at higher latitudes in Norway [21], but based on present day population genetic structure, it does not seem that these populations have contributed extensively to the re-colonization of Scandinavia. Instead, genetic and pollen fossil data suggest that Scandinavia was primarily recolonized from eastern refugia and that Norway spruce reached southern Sweden a few thousand years ago [22]. Interestingly, despite the young age of the Scandinavian Norway spruce populations there is today a strong latitudinal gradient for phenological characters, like bud set and bud flush. This phenotypic gradient has been shown to be largely under genetic control and estimates of heritability have in general been high (above 0.5, [23], [24].

The large and highly heritable variation in growth rhythm responses among populations of Norway spruce can be mainly attributed to differences in reaction to altered photoperiod [23], [25]–[27]. The specific gene variants controlling this divergent response are not known, but a recent study in *P. abies*, using sequence homologs to photoperiod genes from Arabidopsis, identified a number of SNPs showing latitudinal clines in allele frequency across Scandinavia [27]. In particular, SNPs from the promoter of PaFTL2, an FT homolog, and variation in the coding part of PaGI, a GI homolog, are promising candidate SNPs for bud set control. These two genes fit well with observations from gene expression studies in spruce species, where genes related to the photoperiod pathway have been associated with phenology and seasonal growth rhythm [13], [26]–[31].

In the present study, we used two approaches to identify sequence variation in photoperiod pathway genes that significantly deviates from neutral sequences not subjected to selection. First, we tested whether polymorphism and divergence data were consistent with neutral expectations using a maximum likelihood version of the HKA test [32], [33]. Second, we tested for departure from the standard neutral model at photoperiod pathway genes while controlling for demographic history with an Approximate Bayesian Computation (ABC) approach, where background loci were used to fit simple demographic models and the photoperiod pathway genes tested against these scenarios. Genes departing significantly at summary statistics from all tested demographic models were considered to be demographically robust outliers [34] and likely subjected to selection. Interestingly, both methods identified genes deviating from neutral expectations, but not the same genes nor the same part of the photoperiod pathway.

## Results

### Photoperiod pathway genes in Norway spruce

Putative photoperiod pathway genes were identified in EST databases from different spruce species using Arabidopsis photoperiod pathway protein sequence in BLAST searches. Extension of the EST sequences to full-length or near full-length gene sequences from Norway spruce was done using rapid amplification of cDNA ends (RACE). All the sequenced photoperiod genes show strong similarity to photoperiod pathway related genes from flowering plants (Table 1). For most sequences we identified outgroup sequences from both spruce (*P. glauca*, *P. breweriana*, *P. sitchensis*) and pine (*Pinus taeda*), either by amplification and sequencing using the same primers as in Norway spruce or by searching publicly available sequence databases (http://www.plantgdb.org, http://dendrome.ucdavis.edu/). For a subset of the photoperiod pathway genes there are expression and/or functional data that supports them having a role in response to photoperiod [11], [31], [35]

**Table 1:** Annotation of putative photoperiod pathway genes from spruce when compared to the proteins of the model plant Arabidopsis and accession number for the best hit in the current version of the *P. abies* genome sequence.

Gene	AA^1^	FL^2^	Hit A. thaliana^3^	Hit P. abies^4^	Category
PaCRY	259	No	AT4G08920 ATCRY1, cryptochrome 1	MA_10428291	Photoreceptor
PaPHYN-r1	253	No	AT2G18790 PHYB, HY3	MA_73153	Photoreceptor
PaPHYN-rII	229	No	AT1G09570 PHYA, FHY2	MA_73153	Photoreceptor
PaPHYO	437	No	AT1G09570 PHYA, FHY2	MA_6809	Photoreceptor
PaPHYP-rI	264	No	AT2G18790 PHYB, HY3	MA_10435530	Photoreceptor
PaPHYP-rII	91	No	AT2G18790 PHYB, HY3	MA_10435530	Photoreceptor
PaPAT1	70	No	AT5G48150 PAT1	MA_10432093	Photoreceptor
PaZTL	376	No	AT5G57360 Adagio protein 1, ZTL	MA_70291	Photoreceptor
PaGI	115	No	AT1G22770 GI, gigantea protein	MA_19575	Circadian Clock
PaPRR1	558	Yes	AT5G61380 APRR1	MA_71728	Circadian Clock
PaPRR3	168	No	AT2G46670 CCT motif family protein	MA_10316458	Circadian Clock
PaPRR7	290	No	AT5G02810 APRR7	MA_124244	Circadian Clock
PaEBS	139	No	AT4G22140 EBS, early bolting short days	MA_10430427	Downstream target
PaCOL1	410	Yes	AT5G24930 ATCOL4, constans-like 4	MA_54929	Downstream target
PaCOL2	361	Yes	AT5G24930 ATCOL4, constans-like 4	MA_7292	Downstream target
PaMFT1	87	No	AT1G18100 MFT, E12A11	MA_4742	Downstream target
PaMFT2	157	No	AT1G18100 MFT, E12A11	MA_66653	Downstream target
PaFTL1	172	Yes	AT1G65480 Flowering locus T	MA_400747	Downstream target
PaFTL2	66	No	AT5G03840 Terminal flower 1	MA_5386467	Downstream target

1 Number of amino acids available from *Picea abies* used in the protein search

2 Is the sequence a putative full length protein sequence

3 The hit reported is the protein with the lowest e-value when the spruce protein sequence is used as query with the program blastp against the complete protein space of *Arabidopsis thaliana*

4 The hit reported is the best hit obtained with blastn against the gene containing scaffold of the spruce genome assembly v. 1.0 (http://congenie.org).

### Patterns of nucleotide diversity and divergence

In total the analyzed data set contained around 34,000 aligned nucleotides (close to 40% of these are previously unpublished sequence data) from both photoperiod pathway genes and background loci. The average number of aligned sequences across loci was 50 and we identified 750 polymorphic sites over all genes, of which more than one third were singletons (Table 2). The average pairwise nucleotide diversity of the background genes (0.0031) was slightly higher than what was found for the candidate genes (0.0028), despite the fact that candidate genes contained more introns and non-coding sites. The average Tajima's D values were very similar between background (−0.83) and photoperiod pathway related genes (−0.85). Classifying the genes according to their putative position in the photoperiod pathway (see materials and methods for details) shows a pattern where genes assigned as photoreceptors had the lowest level of diversity (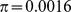) and genes in the circadian clock (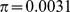) and downstream targets (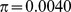) had a mean diversity similar to the mean diversity of background genes. The average non-synonymous diversity was, as expected, lower than both synonymous and silent diversities, but variation around the mean was high and the ratio between non-synonymous and synonymous variation ranged from 0 to 0.81 (Table 2).

**Table 2:** Diversity statistics for the 14 background loci (at the top of the table) and the 19 photoperiodic pathway loci used in the study.

Locus	N^1^	Sites^2^	H^3^	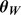 4	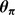 5	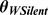	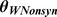
Pa1100	40	346	0.83	0.0041	0.0039	0.0054	0
Pa1151	49	480	0.69	0.0037	0.0021	NA^6^	NA
Pa121	41	440	0.23	0.0021	0.0005	NA	NA
Pa129	49	275	0.47	0.0016	0.0018	NA	NA
Pa1358	49	447	0.68	0.0040	0.0029	0.0097	0.0025
Pa1364	47	552	0.42	0.0016	0.0013	0.0028	0
Pa1368	47	429	0.20	0.0026	0.0010	0.0027	0.0026
Pa1390	49	495	0.92	0.0059	0.0048	0.0111	0.0029
Pa1391	47	503	0.30	0.0018	0.0010	NA	NA
Pa1420	49	571	0.95	0.0082	0.0068	0.0212	0.0028
Pa225	48	209	0.58	0.0065	0.0034	0.0087	0
PaSb16	46	757	0.77	0.0078	0.0044	0.0113	0.0000
PaSb29	46	532	0.85	0.0056	0.0060	0.0050	0.0035
PaSb62	35	537	0.76	0.0063	0.0030	0.0099	0.0011
PaPhyN-rI	54	759	0.62	0.0023	0.0012	0.0051	0.0015
PaPhyN-rII	35	689	0.16	0.0007	0.0002	0.0016	0.0005
PaPhyO	44	1776	0.91	0.0025	0.0016	0.0042	0.0011
PaPhyP-rI	49	794	0.51	0.0011	0.0011	0.0035	0.0004
PaPhyP-rII	53	273	0.44	0.0040	0.0020	0.0106	0.0021
PaCry	52	918	0.42	0.0010	0.0006	0	0.0015
PaPAT1	40	420	0.40	0.0017	0.0020	0.0018	0.0015
PaZTL	41	1220	0.96	0.0063	0.0042	0.0175	0.0009
PaGI	48	772	0.55	0.0020	0.0013	0.0022	0.0019
PaPRR1	32	3939	0.97	0.0059	0.0058	0.0068	0.0041
PaPRR3	42	891	0.78	0.0026	0.0039	0.0037	0.0018
PaPRR7	43	1503	0.72	0.0029	0.0016	0.0039	0.0017
PaCol1	46	3196	0.98	0.0054	0.0030	0.0074	0.0003
PaCol2	71	1191	0.93	0.0066	0.0038	0.0116	0.0042
PaEBS	50	730	0.48	0.0049	0.0023	0.0014	0.0077
PaFTL1	67	2464	0.97	0.0060	0.0036	0.0068	0.0017
PaFTL2	63	644	0.64	0.0056	0.0049	0.0068	0.0014
PaMFT1	70	3997	1.00	0.0090	0.0068	0.0087	0.0000
PaMFT2	90	975	0.95	0.0063	0.0038	0.0096	0.0000

1 Total number of *Picea abies* sequences

2 Number of sites after excluding gaps and sites with missing data

3 Haplotype diversity

4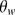: Estimate of the population mutation rate 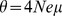, based on the number of segregating sites (per base pair)

5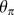: Estimate of the population mutation rate, 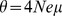, based on nucleotide diversity, 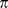. (per base pair)

6 NA = Not applicable

### HKA test

The HKA test compares within-species diversity with between species divergence under a simple split model. Here we used *Pinus taeda* as outgroup and tested three groups of genes (photoreceptors, circadian clock genes and downstream targets) for deviation from the neutral expectation. Only photoreceptor genes showed higher than expected diversity within Norway spruce conditioning on the level of divergence from *P. taeda* (Table 3). This deviation can be largely attributed to the excess of diversity within Norway spruce (33 SNPs) and only 49 differences compared to *P. taeda* at the gene PaZTL, but there were also genes in this group showing low diversity compared to divergence.

**Table 3:** Likelihood values from the mlHKA test of the different group of photoperiodic genes.

Model	Photoreceptors (8)^1^	Circadian clock genes (4)	Downstream targets (4)
Neutral	130.443	104.235	107.351
Selected	120.733	104.361	105.647
Test statistics	19.42	−0.252	3.408
P-value	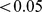	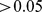	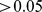

1 The value within parentheses is the degrees of freedom in the likelihood ratio test.

### Demographic inference and detection of outliers among photoperiod pathway genes

We used 14 loci a priori assumed not to be involved in local adaptation or subjected to selection, to infer demographic parameters using an ABC framework. Over the 14 loci, 138 SNPs were identified and close to half of them were singletons. Comparing the ratio of 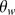 and 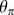 for non-synonymous (1.48) and synonymous (1.46) sites at these loci revealed no major differences in how singletons are distributed between the two classes, justifying the use of all sites to infer demographic history. Three different demographic scenarios were evaluated: the standard neutral model (SNM), a population expansion model (PEM), and finally a more complex demographic scenario that aimed at capturing some of the main features of the demographic history of the species (SPM, Figure S1). This model stems largely from the demographic model proposed by [14] (without Romania due to the low sample size of this population), but rather than treating the two main geographic domains separately, we modelled an ancient bottleneck followed by a split into two main domains and allowed for gene flow between them after the split.

Approximate posterior distributions for the estimated parameters under all models are shown in Figures S2–S4. Under the SNM and the PEM all parameters except 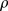 showed fairly narrow distributions. For the more parameter-rich SPM model, parameters were difficult to estimate and their distribution did not show a clear mode. It should be noted that our main goal was not to propose a new demographic model for Norway spruce, but rather to test patterns of nucleotide variation at candidate genes against, not only the standard neutral model, but a set of plausible and more realistic demographic scenarios.

Simulations from the posterior distribution of the SNM identified five genes with a Tajima's D value lower than the expected demographically adjusted 5% quantile and one gene (PaPRR3) with a Tajima's D in the upper 5% quantile (Table 4). For Fay and Wu's H, five outliers were identified. In most cases the deviating patterns were only found for one of the outgroup sequences used. For the PEM, PaPRR3 was the only outlier for Tajimas D and 9 genes showed a significant departure for Fay and Wu's H. Finally in SPM, PaPRR3 was also the only outlier for Tajima's D and six genes showed departure for Fay and Wu's H. The fairly large number of loci deviating under all models for Fay and Wu's H would suggest that none of the three models actually captured all aspects of the demographic history of the species and that the choice of the outgroup sequence also have an impact on the results. Since none of the genes deviated for Fay and Wu's H for all three models and both outgroups used, we took a conservative approach and did not consider any of the analyzed genes as a robust outlier from neutral expectations for this summary statistics. In summary, only PaPRR3 departed significantly for all three models and is the only gene that can be considered a demographically robust outlier that likely has been subjected to selection.

**Table 4:** Test statistics for deviation from neutral expectations for the photoperiod pathway related genes.

Locus	Tajima's D	Fay & Wu's H^a^	Fay & Wu's H^b^	K value from mlHKA
PaPhyN-rI	−1.27	−1.89^2^	0.55	1.49
PaPhyN-rII	−1.28	0.16	NA	0.44
PaPhyO	−1.16	−9.35^1^^,^ 2 ^,^ 3	0.21	0.92
PaPhyP-rI	0.04	0.40	−0.30	0.82
PaPhyP-rII	−1.18	0.36	−1.42^2^^,^ 3	2.42
PaCry	−0.93	−2.92^2^^,^ 3	−4.88^1^^,^ 2 ^,^ 3	0.36
PaPAT1	0.35	−0.72	0.56	0.65
PaZTL	−1.19	−2.72^2^	3.60	4.60
PaGI	−1.00	0.66	0.78	0.90
PaPRR1	−0.09	0.71	NA	1.37
PaPRR3	1.44^1^^,^ 2 ^,^ 3	−0.58	−0.85	0.75
PaPRR7	−1.49^1^	−1.02	−0.29	1.29
PaCol1	−1.57^1^	−13.26^1^^,^ 2 ^,^ 3	−3.38	1.53
PaCol2	−1.37	0.31	−2.97^2^	1.73
PaEBS	−1.67^1^	−9.03^1^^,^ 2 ^,^ 3	−2.61^2^^,^ 3	1.35
PaFTL1	−1.36^1^	NA	NA	NA
PaFTL2	−0.40	−5.73^1^^,^ 2 ^,^ 3	NA	0.63
PaMFT1	−0.87	NA	NA	NA
PaMFT2	−1.21	NA	NA	NA

a With Pinus taeda as outgroup

b With Picea species as outgroup

1 Observed value in the 5% lower or 95% upper quantile for SNM.

2 Observed value in the 5% lower or 95% upper quantile for PEM.

3 Observed value in the 5% lower or 95% upper quantile for SPM.

## Discussion

Genes in the photoperiod pathway have been shown to be implicated in adaptation to local light conditions in several plant species (*e.g.* [1], [36]–[39]). Forest tree species in temperate regions generally show strong latitudinal clines for growth cessation and bud set in response to photoperiod [28], [40]–[42] and we would therefore expect selection to have influenced nucleotide variation at some of the genes from the photoperiod pathway in Norway spruce. In the present study, as well as in a previous one [27], we did indeed detect signatures of selection at some of those genes. However, in spruce, as well as in other tree species, the identity of the genes at which selection was detected seems to strongly depend on the method and the sampling scheme used to detect selection.

Two different approaches were used in this study to detect selection in genes from the photoperiod pathway: first we used the HKA test and second we tested for departures of Tajima's D and Fay and Wu's H statistic from the distribution of these two statistics under different demographic models. In both cases, the analysis was based on a range-wide sample. In contrast to the study by [27], which included SNPs from most of the genes that were used here, there was no attempt to consider a more local geographical scale as sample sizes at local levels were low.

The multilocus HKA test suggests that the diversity at photoreceptor genes is higher than expected considering their level of divergence from *P. taeda*. This significant result is strongly influenced by the relatively high variability of the blue light receptor PaZTL, which has 33 SNPs in Norway spruce and just 49 differences to *Pinus taeda*. There are a number of assumptions underlying these results. In particular, the classification of the genes in the pathway relies on two main assumptions: (i) gene function, and hence classification is conserved between angiosperms and gymnosperms and, (ii) it is meaningful to assign genes to a single position in the pathway and thereby to one of the three groups that we defined a priori. The first assumption may not be as farfetched as it seems, since many photoperiod pathway genes are conserved even in a more distantly related moss species [10] and expression data and functional data for a subset of these genes in spruce do indicate that they might have similar roles as in angiosperms [11], [26], [29]. Based on the results of [31] it appears that PaMFT1 and PaMFT2 group with a clade where functionally characterized genes are involved in embryo development in angiosperms and the expression pattern of the spruce homologs supports a similar role also in spruce. We still keep them as potential downstream targets of the photoperiod pathway in spruce, as this group of genes is highly conserved and minor changes in the protein sequence can lead to functional divergence [31], [39].

Assigning genes to a single position in the pathway is undoubtedly a bit arbitrary given our lack of precise knowledge on the function of spruce photoperiod genes. Further, even in model species some genes are difficult to unambiguously assign to specific pathways. For instance, ZTL represents such a gene as it has been characterized both as photoreceptor and as related to the circadian clock. This ambiguity seems also true in Norway spruce since the spruce homolog PaZTL studied here does not show a diurnal expression pattern under natural light conditions, but Arabidopsis plants overexpressing PaZTL show altered circadian response [11].

To facilitate comparison of our results with the poplar photoperiod pathway, we largely followed the grouping used by Hall and colleagues' [43] study of 25 photoperiod pathway genes in *Populus tremula*. Contrarily to the situation in *P. abies*, genes from this pathway had a lower diversity than control genes in *P. tremula*, but like in *P. abies*, only a few genes departed from neutrality and there was no enrichment of outliers in any of the four gene categories. One of the genes that departed from neutrality in *P. tremula* is the photoreceptor PhyB, which had been previously shown to be implicated in bud set response [44]. There was weaker overlap between the present study and the related results from [27], although signs of selection were detected in PaPRR3 when studying adaptive variation in photoperiod related genes in *P. abies* as well. Also, in both spruce ([27] and this study) and poplar [43], as well as in Arabidopsis (*e.g.* [45], it has been difficult to predict a priori which group of genes in a pathway would show the strongest signal of natural selection. Here we find, as in [45], that earlier acting genes exhibited evidence of non-neutral evolution. However, in poplar the highest values of the scaled selection coefficient for genes were related to the circadian clock rather than to photoreceptors [43]. These seemingly contrasting results probably reflect the rather arbitrarily nature of pathways and the fact that genes are often highly pleiotropic. This can be nicely exemplified with the recent finding that the flowering time gene FLC binds to around 780 genes involved in diverse processes [46].

Using an ABC approach we also evaluated the pattern of diversity of photoperiod pathway genes under three different demographic scenarios. Heuertz [14] proposed an ancient and severe bottleneck followed by population expansion as the most likely demographic scenario based on multilocus patterns of Tajima's D and Fay and Wu's H values. Here we used partly the same data and used two simple standard models as well as a more complex model largely capturing the properties of the demographic history proposed by [14]. As multilocus sequence data has become easier to obtain in a number of studies on plants with large natural distribution ranges, it has become clear that most species deviate strongly from the standard neutral model. As mentioned already in the introduction, the timing of inferred bottlenecks from European tree species suggests that the bottleneck does not correspond to recent glaciation events, but appears to be older. However, the exact timing of these events depends on a number of assumptions, such as mutation rate and generation time, creating a large confidence interval for both the timing and severity of bottlenecks. Besides, none of the three models are likely to capture all aspects of Norway spruce past demographics so we used departure from the three models as a benchmark for selection. It would be premature to make a definitive choice regarding demographic scenario on the basis of currently available sequence data, since the number of loci studied is still limited and only the gene space has been explored. Furthermore, pooling data from the complete distribution range of a species with population genetic structure, can under specific scenarios lead to a skew in the observed frequency spectrum and hence affect summary statistics like Tajima's D, even though the effect on smaller scale data sets like ours might not be extensive [47], [48]. Any detrimental effect of pooling here, is likely to be limited as the most complex model (SPM) includes both population subdivision and growth and would hence incorporate the effect of pooling. Only PaPRR3 departed from all three models, with Tajima's D values higher than the simulated data in all cases. The highly positive value is not only an outlier from these tested models, but is also in the very tail of the observed values of Tajima's D values reported from Norway spruce [14], [49]–[51]. This indicates an excess of intermediate-frequency variants and has often been explained by balancing selection. In the present case, the excess of common variants could rather be a consequence of the putative role of PaPRR3 in the response to photoperiod and reflect divergent selection between the northern and southern populations, thereby leading to two main groups of alleles. This is not strong enough to be clearly seen when clustering sequences based on similarity (data not shown), but in earlier studies of SNPs from the same gene there is support for at least one SNP showing a higher than expected 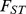 value between populations from different latitudes [27]. This explanation is, however, not fully satisfying as the overall pattern of clinal variation and signs of local adaptation in [27] were stronger for PaPHYP, PaGI, PaPRR7, PaFTL2, genes that do not deviate from neutral expectations here. On the other hand, given that the different neutrality tests consider different time scales and null hypotheses, there is no strong rationale for expecting them to identify the same polymorphisms.

Interestingly, in several domesticated species (*Hordeum vulgare* [38], *Triticum aestivum* [37] and *Beta vulgaris* [39]) PRR homologs were shown to be involved in divergent responses to photoperiod. In these species, mutations have altered sensitivity to photoperiod and both non-synonymous and regulatory changes have been identified and shown to be involved in the response. Hence, it seems that different types of mutations might be able to confer changes in the sensitivity to photoperiod and it will be hard to predict which types of changes are most likely to confer change in sensitivity to photoperiod. Further, the artificial selection associated with domestication and breeding might be quite different from natural selection. We have not sequenced any part of the regulatory region of PaPRR3, but several non-synonymous mutations are present within the coding region. These could alter interactions with other clock genes or photoperiod pathway related genes and hence confer differences in photoperiodic response.

## Conclusions

The large impact of photoperiod genes in local adaptation together with the conservation of such genes over hundreds of millions of years make them excellent candidate genes for adaptation to local light conditions in a wide range of plant species. Here we show that diversity at genes in the photoperiod pathway in Norway spruce is not compatible with neutral expectations and in particular PaPRR3 and PaZTL have likely been subjected to selection. We cannot from the present data pinpoint the nature of the selection that acted on either of the two genes, but the diversity observed in PaPRR3 is at least compatible with a role in local adaptation. Although PaPRR3 was not among the top candidate genes involved in local adaptation in a recent study of clinal variation in Norway spruce [27], it emerged as the most robust candidate in the present study. The outcome of large-scale association studies and expression studies will eventually be needed to resolve the role of photoperiod pathway related genes in local adaptation in Norway spruce.

## Materials and Methods

### Plant material

**Figure 1:**
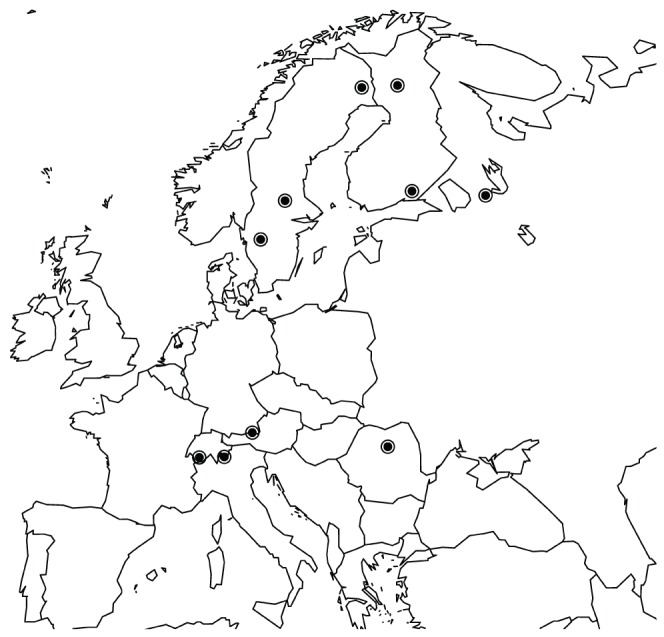
Map of Europe with sample locations shown as dots.

Seeds were collected from 10 locations, either from natural stands of Norway spruce or from seed orchards representing the local population. The sampled populations are distributed throughout a large portion of the natural distribution range (Figure 1). Over all loci and from each population an average of 6 to 7 megagametophytes were sequenced.

### Ethics Statement

Seed samples from the locations used in the study are not from any endangered or protected species and do not require special permits to be collected.

### Molecular methods

DNA was extracted from individual megagametophytes using a slightly modified CTAB procedure or with the DNeasy Plant Mini kit (Qiagen, Valencia, CA). Putative photoperiod genes in Norway spruce were identified from spruce EST sequences, assembled to putative unique transcripts at PlantGDB (http://www.plantgdb.org/, PUT-release 157a) using Arabidopsis photoperiod pathway protein sequences as queries. For a subset of genes full-length cDNA sequences were acquired with rapid amplification of cDNA ends (RACE) following the manufacturers instruction (Clontech, Mountain View, CA). In total 19 photoperiod genes and 14 background genes were amplified and sequenced for 32–90 individuals from the natural distribution range of Norway spruce (Figure 1). The term background gene refers only to the fact that these fragments are not a priori believed to be involved in photoperiodic response. The intron/exon structure was obtained by aligning the resulting genomic sequence to the corresponding cDNA sequence. Alignments of the 14 background genes as well as 11 of the candidate genes were obtained from previous studies [14], [49].

All PCR reactions were made with 100% proofreading Phusion DNA Polymerase (Finnzymes, Espoo, Finland). PCR products were purified with Exo-SAP and directly sequenced from PCR products with either BigDye v3.1 on an ABI 370 or 3730XL (Applied Biosystems, Foster City, CA) or with Dyenamic ET terminators on a MegaBace 1000 (GE Healthcare, Piscataway, NJ). Most regions were covered by two or more reads. Sequences were base-called and assembled with PHRED and PHRAP [52], [53] and visualized and edited with CONSED version 13.0 [54].

### Data analysis

The sequenced fragments were grouped in two main groups, background loci and putative photoperiod pathway loci, where the latter are candidate genes for involvement in photoperiodic response in Norway spruce. The background genes in this study are assumed not to be involved in local adaptation to photoperiod and based on sequence similarity to Arabidopsis none of them show any similarity to genes that have been assigned to the photoperiod pathway in Arabidopsis (data not shown). The photoperiod pathway genes were further grouped according to their putative position in the photoperiod pathway, largely following the grouping that was done in recent study looking at photoperiod pathway related genes in poplar [43]. Three groups were defined: photoreceptors, circadian clock genes and downstream targets (Table 1). This classification was used to test if any particular part of the pathway is under selection using the maximum likelihood HKA test developed by [33]. Under a standard neutral model, within-species diversity should correlate with between species divergence and this test allows identifying genes that display a deviating pattern of diversity compared to divergence. Using all genes where an outgroup (a single sequence of *Pinus taeda*) was available, the program was first run for 1 million steps under a neutral split model and then run for 1 million steps allowing selection at the genes assigned to the three different groups of photoperiod pathway genes defined above while imposing the neutral model on the background loci. Under the selection model a selection parameter k is estimated for focal genes. This k value is larger than one if within species polymorphism is larger than expected under neutrality and lower than one if it is smaller. We performed the HKA test with *Pinus taeda* as outgroup only and not with sequences from other Picea species that were also available because shared polymorphisms are common between spruce species [49], [55], showing that they have not diverged long enough to fulfill the assumptions of the HKA test.

DnaSP v. 5 [56] was used to analyze intra- and interspecific sequence variation. Nucleotide diversity and the proportion of segregating sites were calculated ignoring both indels and sites with missing data.

The Approximate Bayesian Computation (ABC) approach implemented in the software Egglib [57], was used to test for deviations from neutral expectations conditional on demographic scenarios. Three demographic scenarios were considered and the ABC analysis was based on the 14 background loci. The three scenarios were (i) the standard neutral model (SNM) that includes two parameters; the population mutation parameter, 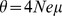, where Ne is the effective population size and 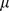 the per-generation per-base pair mutation rate, and 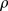, the population recombination parameter, 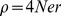, where Ne is the effective population size and r the per-generation recombination rate between adjacent base pairs, (ii) a population expansion model (PEM) with three parameters; 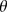, 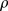, and 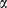, an exponential growth factor, and finally (iii) a more complex split model (SPM) that includes an ancient bottleneck followed by a split into two populations and population expansion. This model has 8 parameters; 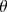 and 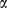 as in the previous model and six additional parameters: M, the migration between the two descendant populations, N1, the size of the first descendant population, and NA, the effective population size for the ancestral and the second descendant population, which are assumed to have the same Ne, T1, the time of population split, T2, the time of the bottleneck and S, the bottleneck severity. A graphical representation of the model can be found in Figure S1. In the SPM model we chose not to include 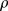, as the number of parameters was already high. Not including 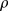 in the model should not strongly skew the results as the background loci are rather short and we therefore have low power to estimate 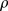. Second, ignoring recombination makes tests of selection based on the site frequency spectrum more conservative. The number of segregating sites, Tajima's D [58] and Fay and Wu's H [59] were used as summary statistics to fit the first two demographic models. The ancestral states of polymorphic positions were inferred by using a single sequence of *Pinus taeda* and/or a single sequence from any of the species *Picea glauca*, *P. sitchensis* or *P. breweriana* when available. Six summary statistics were used in the SPM model: 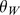, 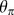 and He were used to characterize polymorphism within populations and 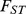 [60], 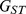 [61], and Snn [62] to characterize population divergence; wide uniform priors were used for all parameters and 10 million data points were simulated from which 1% of the values were retained and used for regression of parameter values.

To test the photoperiod pathway genes against the demographic scenarios inferred from the background loci we randomly sampled 10,000 data points from the inferred posterior distribution of each of the models and calculated the expected distributions of Tajima's D and Fay and Wu's H values. Observed values of these summary statistics were calculated for the candidate genes, using the outgroups for Fay and Wu's H as described for the background loci. We then tested empirically if the observed Tajimas D and Fay and Wu's H values departed from their expected values by estimating the 5% confidence intervals with the R package Boa [63]. The latter allows for approximate estimation of confidence intervals for posterior distributions.

## Supporting Information

Figure S1 **Cartoon of the complex split and growth model (SPM).**
(PDF)

Figure S2 **Density plots of parameters estimated with ABC using the Standard Neutral Model (SNM).**
(PDF)

Figure S3 **Density plots of parameters estimated with ABC using the Population Expansion model (PEM).**
(PDF)

Figure S4 **Density plots of parameters estimated with ABC using the split and growth model (SPM).**
(PDF)
