# Supplementary figures and images for: Patterns of Nucleotide Diversity at Photoperiod Related Genes in Norway Spruce [Picea abies (L.) Karst.]
Source: PLoS One. 2014 May 8;9(5):e95306. doi: 10.1371/journal.pone.0095306 (PMC4014479; doi:10.1371/journal.pone.0095306)

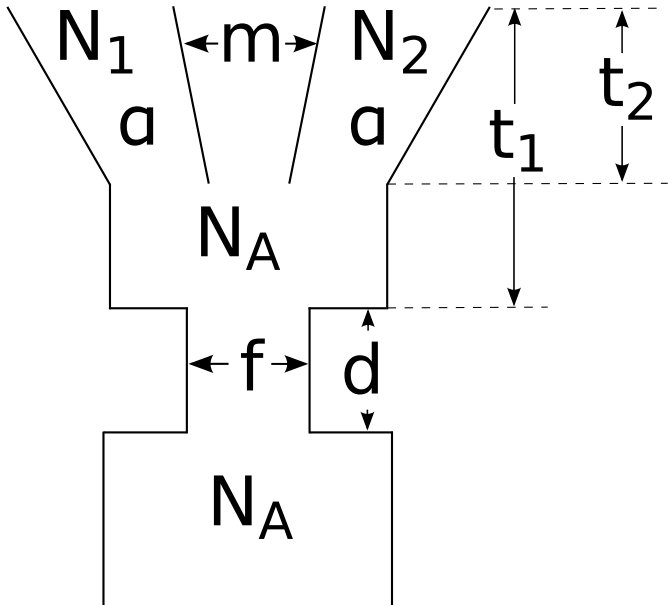

Supplement: Figure S1 — Cartoon of the complex split and growth model (SPM). (PDF) [file pone.0095306.s001.pdf]

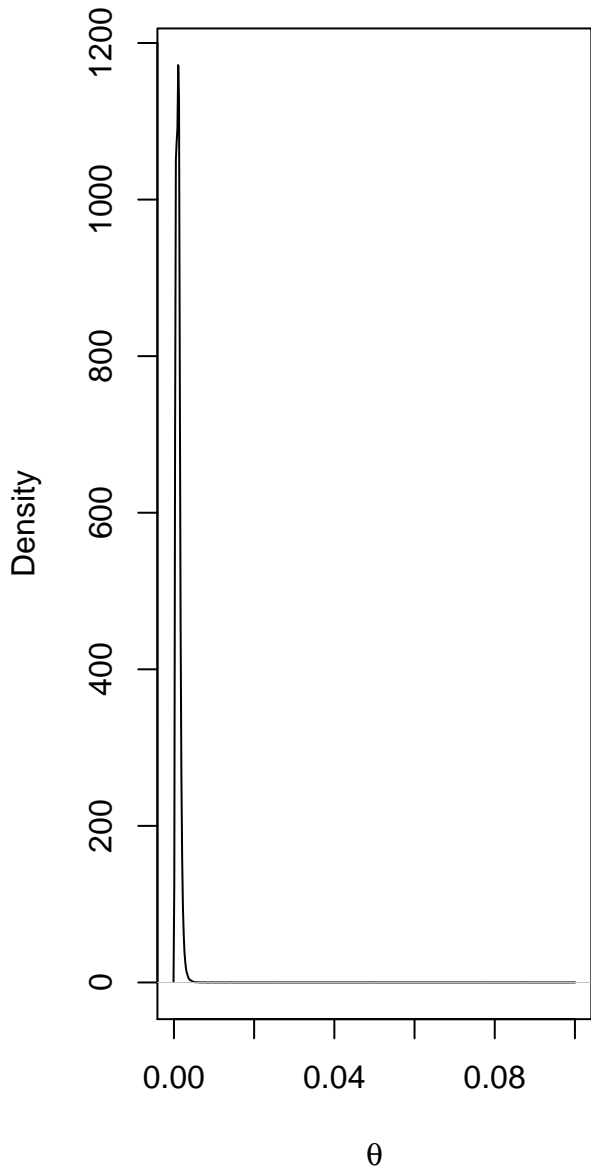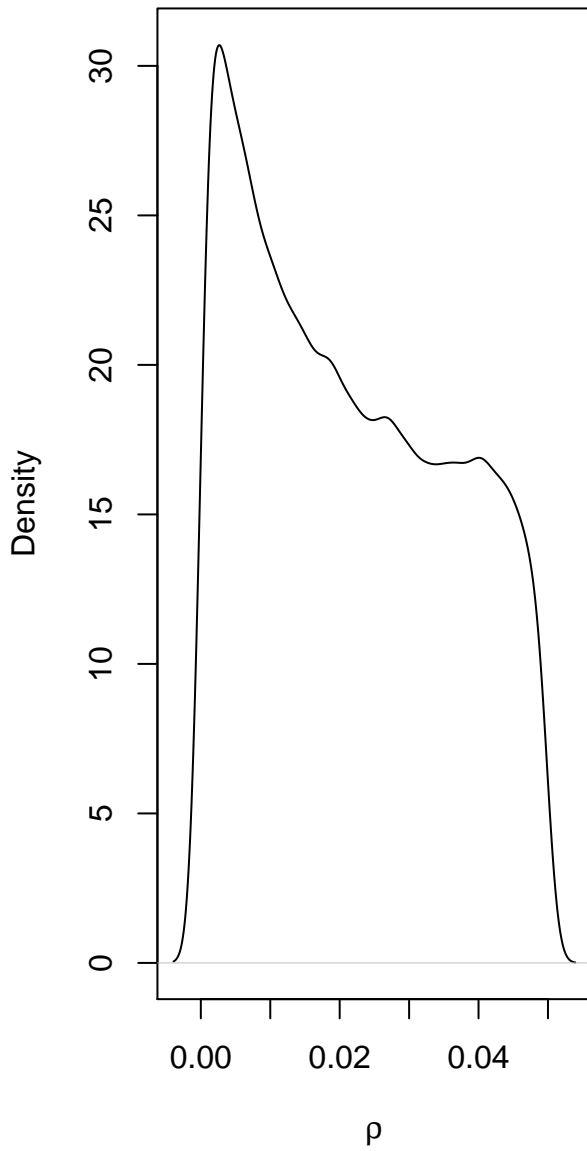

Supplement: Figure S2 — Density plots of parameters estimated with ABC using the Standard Neutral Model (SNM). (PDF) [file pone.0095306.s002.pdf]

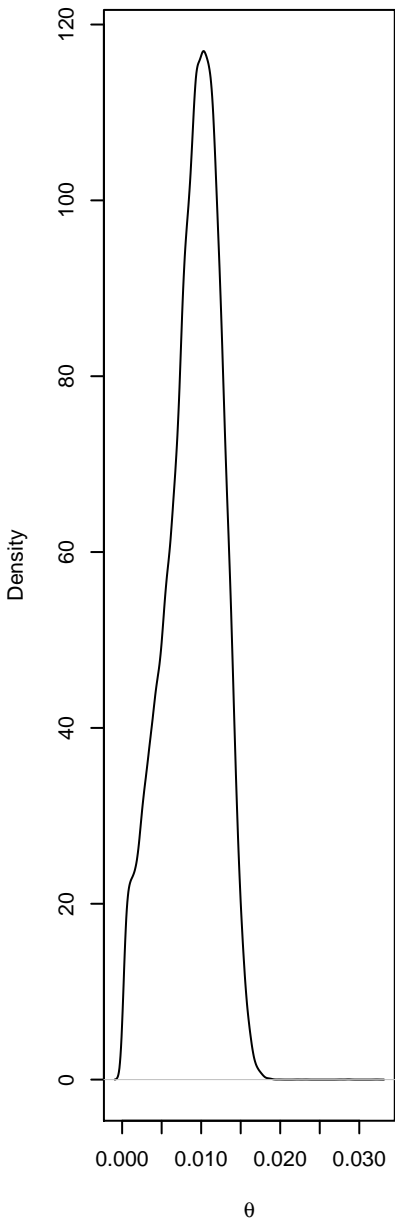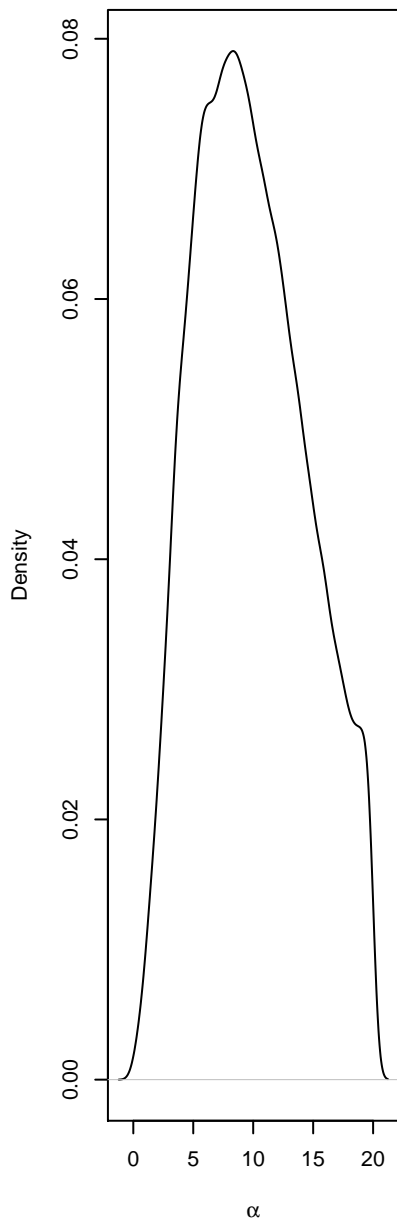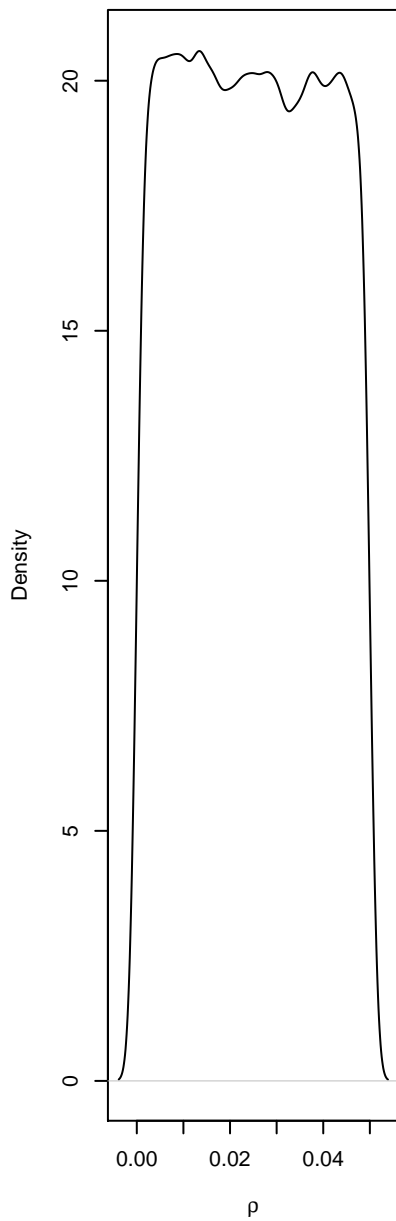

Supplement: Figure S3 — Density plots of parameters estimated with ABC using the Population Expansion model (PEM). (PDF) [file pone.0095306.s003.pdf]

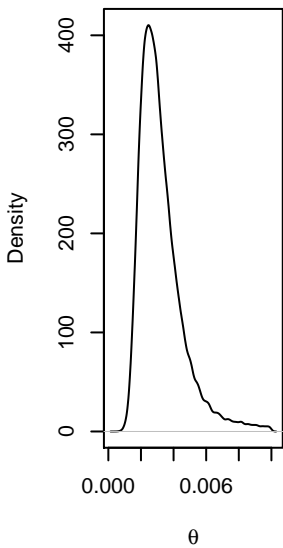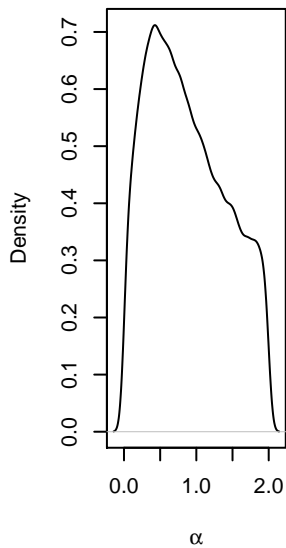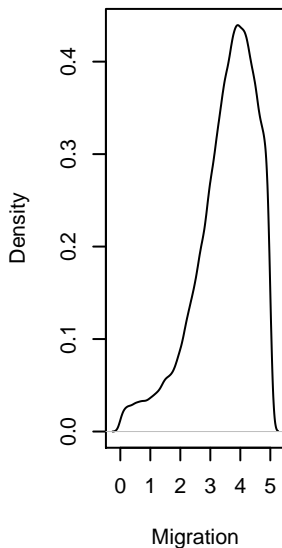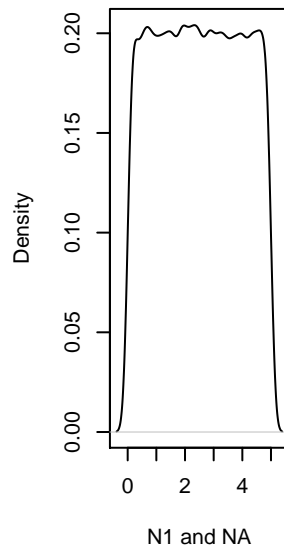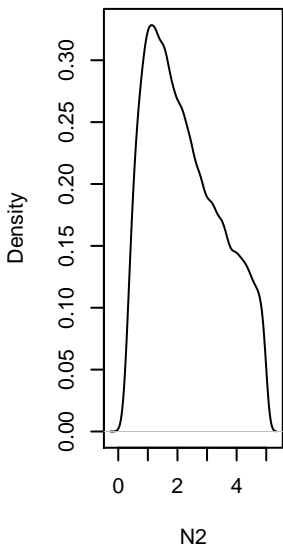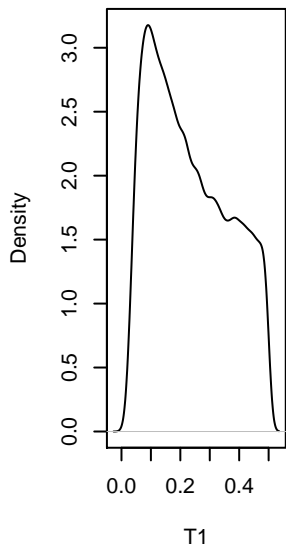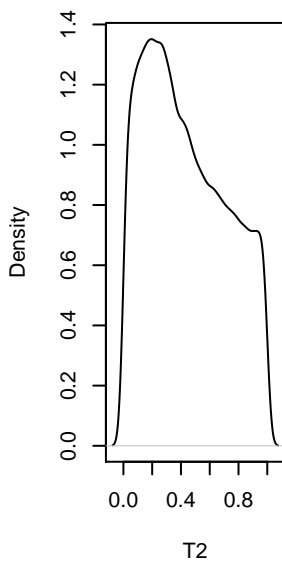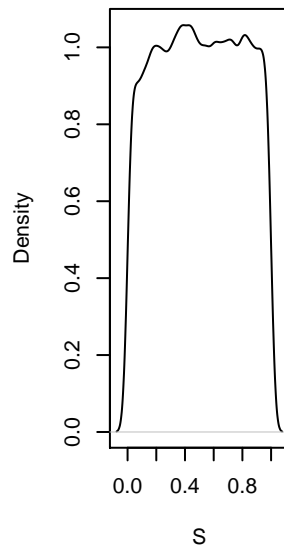

Supplement: Figure S4 — Density plots of parameters estimated with ABC using the split and growth model (SPM). (PDF) [file pone.0095306.s004.pdf]
